# Can contracted out health facilities improve access, equity, and quality of maternal and newborn health services? Evidence from Pakistan

**DOI:** 10.1186/s12961-015-0041-8

**Published:** 2015-11-25

**Authors:** Shehla Zaidi, Atif Riaz, Fauziah Rabbani, Syed Iqbal Azam, Syeda Nida Imran, Nouhseen Akber Pradhan, Gul Nawaz Khan

**Affiliations:** Department of Community Health Sciences, Aga Khan University, P.O. Box 3500, Stadium road, Karachi, 74800 Pakistan; Women and Child Health Division, Aga Khan University, P.O. Box 3500, Stadium road, Karachi, 74800 Pakistan

**Keywords:** Contracting out, Maternal and newborn health, Public health facilities

## Abstract

**Background:**

The case of contracting out government health services to non-governmental organizations (NGOs) has been weak for maternal, newborn, and child health (MNCH) services, with documented gains being mainly in curative services. We present an in-depth assessment of the comparative advantages of contracting out on MNCH access, quality, and equity, using a case study from Pakistan.

**Methods:**

An end-line, cross-sectional assessment was conducted of government facilities contracted out to a large national NGO and government-managed centres serving as controls, in two remote rural districts of Pakistan. Contracting out was specific for augmenting MNCH services but without contractual performance incentives. A household survey, a health facility survey, and focus group discussions with client and spouses were used for assessment.

**Results:**

Contracted out facilities had a significantly higher utilization as compared to control facilities for antenatal care, delivery, postnatal care, emergency obstetric care, and neonatal illness. Contracted facilities had comparatively better quality of MNCH services but not in all aspects. Better household practices were also seen in the district where contracting involved administrative control over outreach programs. Contracting was also faced with certain drawbacks. Facility utilization was inequitably higher amongst more educated and affluent clients. Contracted out catchments had higher out-of-pocket expenses on MNCH services, driven by steeper transport costs and user charges for additional diagnostics. Contracting out did not influence higher MNCH service coverage rates across the catchment. Physical distances, inadequate transport, and low demand for facility-based care in non-emergency settings were key client-reported barriers.

**Conclusion:**

Contracting out MNCH services at government health facilities can improve facility utilization and bring some improvement in  quality of services. However, contracting out of health facilities is insufficient to increase service access across the catchment in remote rural contexts and requires accompanying measures for demand enhancement, transportation access, and targeting of the more disadvantaged clientele.

## Background

Contracting out involves a formal agreement between a government, as the financier, and a private sector or autonomous government provider for a mutually agreed set of services, in a specified location, over a defined period [1]. Insufficient access to maternal, newborn, and child health (MNCH) services is one of the reasons for the high mortality surrounding births [2]. Contracting out of government-provided health services to NGOs is an increasingly popular means to increase access to health services in remote areas where there is little government capacity to provide such services and is based on a stipulated contract agreement and targets. It has been applied in fragile states, such as Afghanistan and Cambodia, for quick roll out of service delivery as well as more stable states, such as Bangladesh, India, and Pakistan, for improved delivery of health services [3].

Existing evidence suggests that contracting healthcare services has resulted in increased utilization of primary healthcare services [4,5], but good quality evidence is scarce on improvement in MNCH service utilization. Moreover, even where contracting has successfully increased primary healthcare utilization, there remain critical knowledge gaps. First, for example, little is known about equitable penetration of benefits to more disadvantaged groups, such as those who are poorer, less educated, or living at further distance from health facilities, and hence bear the major burden of maternal and infant mortality. Second, there is meagre information as to whether contracting, while increasing health service utilization, also reduces out-of-pocket (OOP) expenditure borne by patients. Third, there is little evidence on client-perceived barriers to using contracted facilities as available evidence tends to focus on quantitative assessments, neglecting community perspectives.

Herein, we examine the relevance of contracting out health services to NGOs to augment government health facilities and services for MNCH. An attempt is made to fill critical knowledge gaps through a case study of contracting out from Pakistan. In Pakistan, there has been a mushrooming of contracting initiatives in recent years, involving the contracting of government health facilities to large NGOs in rural settings. However, expansion of contracting out schemes has taken place without being accompanied by independent monitoring and evaluations. This paper presents an in-depth assessment of the comparative advantages, if any, of contracted out facilities versus government-managed facilities in remote rural settings. These are compared in terms of (1) utilization of facility and outreach services; (2) equitable utilization by the disadvantaged; (3) quality of services; and (4) patient expenditure. In addition, client-perceived barriers that affect the use of contracted versus government-managed facilities for MNCH services are explored. The paper concludes with a synthesis of how contracting of government health facilities can be strengthened for MNCH in rural disadvantaged settings based on lessons learnt from the experience of Pakistan.

### Setting

#### The healthcare system

Pakistan is the sixth most populous country in the world, with an estimated population of more than 165 million; 12.4% of its population lives below the poverty line [6] and 42% are illiterate [7]. Pakistan lags behind neighbouring countries in key health indicators, including the maternal mortality ratio of 276/100,000 live births and the neonatal mortality rate of 54/1000 live births [8]. There is a mixed health system in Pakistan, with co-existing public and private sectors. The public sector has a three-tiered infrastructure for service delivery which includes 965 tertiary and secondary hospitals, 581 Rural Health Centres (RHCs), and 5798 Basic Health Units (BHUs) for primary healthcare services [9]. The RHC typically serves a catchment of 20,000 to 50,000 population providing facility-based primary services, limited secondary services, and oversees outreach programs including the Lady Health Workers (LHWs) program, malaria, and Tuberculosis Directly Observed Treatment, Short course programs. MNCH services provided at the RHC include basic emergency obstetric and neonatal care (BEmONC), inclusive of complicated (non-caesarean) deliveries and sick child management, as well as routine services such as antenatal care (ANC), antenatal diagnostic workup, delivery, postnatal care (PNC), family planning, immunizations, integrated management of childhood illness, and growth monitoring.

#### Organizational and policy changes

In Pakistan, an extensive contracting initiative – the People’s Primary Healthcare Initiative – is already in place, whereby 2,392 BHUs across the country have been contracted out to a national NGO [10]. A third party evaluation of this initiative suggests an increase in facility-based curative care utilization, but limited improvement in facility-based utilization of MNCH services [10]. In a major constitutional change, the provincial legislation now supersedes federal legislation for the health sector, empowering provinces to introduce alternative ways of service delivery in response to contextual needs. Extensive contracting out beyond the BHUs is an option under consideration in all four provinces as part of their post-devolution health sector strategies [11,12]. Meanwhile, piecemeal contracting-out of RHCs to local and international NGOs is already in place in three of the four provinces of Pakistan, including Khyber Pukhtunkhwa, Sindh, and Baluchistan [13].

The study was conducted in two contracted-out RHCs, one each from Thatta and Chitral districts, and four matching government-managed RHCs which served as controls, with an intervention to control ratio of 1:2. The contracted RHCs were located in far flung and underdeveloped Talukas (sub-district level); Keti Bunder in district Thatta and Shagram in district Chitral, with a distance of 60 km or more from a RHC to the next government health facility. The control RHCs from both Thatta and Chitral were selected with participation of respective district health offices and objectively based on comparable catchment population size, BCG vaccine coverage, number of LHWs working in RHC catchments, and geographical location such as proximity to road or town centre.

## Methods

### Contractual intervention examined

The RHCs assessed had been contracted out to a national NGO for provision of MNCH services since 2008. The RHC tier was targeted as it has not been previously explored, allows evaluation of BEmONC as well as routine MNCH services, and is of policy relevance due to continuous expansion of contracting to RHCs. The contractual package included provision of facility-based routine and BEmONC services. In Chitral, the contract additionally stipulated the provision of caesarean delivery as well as administrative control of outreach preventive care programs. The contracts were based on a block grant and did not include specific targets for the agreed service package. Formal controls brought in by the contract included (1) posted government staff made available to the NGO but without transfer and termination authority; (2) additional NGO- supported staff hired with incentivised salary; (3) introduction of an expanded number of essential drug categories and diagnostic tests; (4) authority over maintenance of building and equipment related to MNCH services; (5) introduction of user charges for additional diagnostics that were not covered by RHC budget; and (6) introduction of user charges for antenatal and delivery registration.

### Study design

An end-line, cross-sectional comparison was conducted between contracted RHCs and their designated catchment areas and matching control sites using a case study design. Mixed methods were used to bring together a comprehensive account of case of contracting out [14], which has thus far been neglected in previous studies. In addition, we aimed to ensure that findings from different methods were mutually corroborated to strengthen the argument [15]. These included a household survey, a health facility assessment using the Balanced Scorecard (BSC), and focus group discussions (FGDs). Baseline data were not available.

### Household survey

The household survey was carried out in 358 villages in the catchment areas of each of the two contracted and four control RHCs. Catchment villages were stratified into near cluster (distance ≤5 km from the nearest RHC) and far clusters (distance >5 km from the nearest RHC). Within each cluster, the larger villages were divided into household units of 50, while those having less than 50 households were taken as discrete units. Out of 343 household units, a total of 180 units were randomly sampled in both the near and far clusters. Within each sampled household unit, all eligible households who were willing to participate in the survey were interviewed. Sample size was calculated based on 8.2% of births in public sector facilities [8], with the ability to detect at least a 7 percentage point difference in institutional deliveries, 80% power, and 5% level of significance. A total of 8,763 households were visited to sample 1,004 respondents, comprising of 350 in contracted sites and 654 in control sites. To minimize recall bias, the interviews were conducted with women who had delivered in the last 6 months on provider utilization for a range of MNCH services (ANC, facility-based delivery, PNC, care for newborn illness), OOP expenditure, birth outcome, awareness and practices of safe behaviours, and sociodemographic profile. An asset index was computed based on household assets and characteristics and respondents ranked into three tertiles, with I as the richest and III as the poorest tertile. Data from Thatta and Chitral were pooled for performance comparison of contracted and control sites and also separately analysed to assess district differences due to contractual interventions. We also looked at the utilization of contracted and control RHCs by disadvantaged and less disadvantaged groups. Disadvantaged groups were taken as the illiterate, those residing at a distance of >5 km, and those with poorest socioeconomic status, i.e. third tertile. A χ^2^ test and Fisher’s exact test were performed for comparison between contracted and control RHCs. Expenditure data was trimmed for outliers by removing 10% of the data from both tails [16].

### Health facility readiness assessment

The BSC approach was used to assess readiness of two contracted and four control RHCs to deliver quality care. Five domains were assessed: patient satisfaction, staff satisfaction, staff capacity, service provision, and health facility functionality index. Validated Service Provision Assessment tools [17] were adapted and field tested to collect data on 20 indicator categories related to these domains. A combination of data collection methods was used to provide information for feeding into BSC and included checklists for indent review, exit patient interviews, staff interviews, and direct observations. Service provision guidelines of the National MNCH program in Pakistan [18] formed the basis for checking the availability of MNCH and laboratory services, process of care, and the presence of drugs, equipment, supplies, physical infrastructure, health management information system records, and waste disposal systems. Sixty exit interviews with pregnant or recently delivered mothers and parents of newborns were conducted for patient satisfaction and adequacy of provider communication to patients. Thirty six staff interviews – six per facility – were conducted with service delivery and management personnel to assess knowledge, extent of supervision, and staff satisfaction. Thirty six direct observations were made on purposively selected clients availing MNCH services to assess the quality of care and provision of required services as per the National MNCH guidelines. Table 1 illustrates BSC domains and their sources of information. The BSC domains were converted into indices, created from an aggregate set of available performance indicators, and composite scores were calculated based on mean percentages. Composite scores of each domain were finally used to compare the performance of contracted and control facilities.

**Table 1:** **Balanced scorecard domains and tools**

**Domains**	**Tools of data collection**
Patient satisfaction	Client exit interviews
Staff satisfaction and capacity	Staff interviews
Service provision	Direct observations and client exit interviews
Health facility functionality	Facility indent review checklist and direct observations

### Focus groups discussions (FGDs)

Community preferences and barriers to seeking MNCH services at contracted and control RHCs were explored through 24 FGDs with pregnant women or those who had recently delivered, as well as 12 FGDs with their spouses. Six FGDs, four with mothers and two with spouses, were conducted with the catchment population of each participating RHC giving a total of 36 FGDs. FGDs are a useful method of obtaining detailed information as they provide an opportunity for follow-up and probing by facilitator and group [19]. FGDs were conducted in villages of near and far clusters. Equal numbers of villages were randomly selected from a computer generated list. There were 10–14 participants in each FGD. A topic guide with probes, prepared with the help of background literature search, was used by the moderator to initiate a free flow of discussion while two note-takers captured the discussion. Data collectors identified eligible participants from the villages and invited them for FGDs. FGDs were conducted within the villages at convenient times and places for participants. The FGDs began with exploring health seeking behaviour across the range of MNCH services and probing barriers to MNCH service utilization. In addition, decision making dynamics at household level for use of MNCH services were explored. All transcripts were uploaded into QSR NVivo 10.0 software for easy and systematic retrieval of information for analysis. Transcripts were coded into initial codes using a grounded process of issues identified by respondents and then thematically grouped into branch codes [20].

### Ethical considerations

Ethical approval was obtained from the Ethical Review Committee of Aga Khan University Karachi and the National Bioethics Committee of Pakistan. Permission from village community leaders of sampled villages and from medical officers in charge of selected health facilities was obtained prior to data collection, while informed consent was obtained from all interviewees. Names of respondents were anonymized with a code for analysis and writing up and data was secured with restricted password access.

## Results

### Comparability of contracted and control sites

The catchment populations of contracted and control sites were comparable in terms of family size (*P* = 0.18) and maternal literacy (*P* = 0.33). However, participants of contracted catchments were significantly poorer than those in control catchments (*P* <0.001).

### Utilization of MNCH services

Results from the household survey showed that the utilization of contracted RHCs was significantly higher than control RHCs for facility-based births, ANC 3+ visits, PNC, and care for newborn illness. Higher utilization for complicated deliveries is confined to only Chitral, which had a BEmONC package (i.e. 71.1% in contracted RHC vs 3.9% in control facility). However, there was no significant difference across contracted and control catchment areas for overall population-based utilization of services by skilled provider (Table 2).

**Table 2:** **Utilization of MNCH services**

	**Utilization specific to rural health centres**			**Utilization of skilled provider** ^**a**^		
**Parameters**	**Contracted**	**Control**	***P*** **value**	**Contracted**	**Control**	***P*** **value**
	***(n = 350; %)***	***(n = 654; %)***		***(n = 350; %)***	***(n = 654; %)***	
Antenatal care at least one visit	209 (75.5)	148 (26.6)	<0.001	273 (78.0)	539 (82.4)	0.07
Antenatal care three or more visits	106 (82.2)	79 (28.0)	<0.001	129 (36.9)	278 (42.5)	0.31
Facility-based delivery (all types)	81 (23.1)	30 (4.6)	<0.001	200 (57.1)	332 (50.7)	0.07
Facility-based complicated delivery	27 (48.2)	4 (3.3)	<0.001	55 (15.7)	115 (17.5)	0.41
Postnatal care (within 6 weeks of delivery)	17 (29.8)	15 (10.5)	<0.001	49 (14.0)	129 (19.7)	0.38
Care seeking for newborn illness	27 (22.5)	17 (7.9)	<0.001	90 (25.7)	166 (25.4)	0.64

^a^Skilled provider included doctors, nurses, and lady health visitors situated in rural health centres, government facilities other than rural health centres, and private health facilities.

### Equitable utilization of MNCH services

A comparison of utilization pattern across contracted out and control sites showed a significant difference across disadvantaged and less disadvantaged groups in the use of facility-based ANC, but no such appreciable difference was seen in the use of facility-based deliveries. Data for PNC and care seeking for newborn illnesses were sparse. The findings for ANC showed a mixed pattern. The contracted out sites had a significantly higher proportion of literate users and those in the highest socioeconomic tertile, than control sites (*P* <0.001; Table 3). At the same time, a higher proportion of ANC users at contracted facilities were residing more than 5 km away as compared to the control site, where the majority of users were living within the closer radius of <5 km (*P* <0.01).

**Table 3:** **Rural health centre service utilization by disadvantaged groups in contracted out and control catchments**

**Service utilization**	**Contracted out**	**Control**	***P*** **value**
	***n (%)***	***n (%)***	
Antenatal care (n = 357)			
Illiterate	134 (64.1)	133 (89.9)	<0.001
Literate	75 (35.9)	15 (10.1)	
Distance >5 km	130 (62.2)	68 (45.9)	<0.01
Distance <5 km	79 (37.8)	80 (54.1)	
SES tertile I	88 (42.1)	34 (23.0)	<0.001
SES tertile II	52 (24.9)	86 (58.1)	
SES tertile III	69 (33.0)	28 (18.9)	
Facility-based births (n =111)			
Illiterate	54 (66.7)	25 (83.3)	0.10
Literate	27 (33.3)	5 (16.7)	
Distance >5 km	35 (43.2)	9 (30.0)	0.27
Distance <5 km	46 (56.8)	21 (70.0)	
SES tertile I	30 (37.0)	9 (30.0)	0.41
SES tertile II	24 (29.6)	13 (43.3)	
SES tertile III	27 (33.3)	8 (26.7)	

*SES* Socioeconomic status.

### Awareness and practices of safe behaviours

There were more positive preventive health measures seen in contracted catchment than in control sites in Chitral, as compared to Thatta (Table 4). A significantly higher percentage of mothers in contracted catchments of Chitral were aware of one or more danger signs of pregnancy and of newborn complications than control catchments. Similarly, cord handling practices involving safe cutting and tying of cord, BCG immunisation to newborns, early initiation of breastfeeding, use of colostrum, and lesser use of pre-lacteal feeds were significantly better in contracted sites of Chitral as compared to government-managed catchment areas. However, not all indicators were superior, with no difference seen in oral rehydration solution use and tetanus toxoid immunization. In Thatta, no such comparative edge was seen for contracted catchments over control sites and, in fact, mothers in contracted areas had poorer breastfeeding and cord handling practices than in control areas.

**Table 4:** **Differences in awareness and practices of safe behaviours at household level between contracted out and control sites – disaggregated by Thatta and Chitral**

**Parameters**	**Thatta** ***(n = 394)***		**Chitral** ***(n = 610)***	
	**Outreach package not in contract**		**Outreach package in contract**	
	**Percentage difference**	***P*** **value**	**Percentage difference**	***P*** **value**
Women aware of at least one danger sign of pregnancy	8.1	0.08	11.5	<0.01
Women receiving two or more TT injections during last pregnancy	2.8	0.58	−4.8	0.18
Women aware about at least one danger sign of newborn illness	7.5	0.15	8.6	0.02
Safe cord handling practices	−13.8	<0.01	13.4	<0.001
Immunized for BCG at birth	9.2	0.09	10.6	<0.01
Newborns weighed at birth	28.8	<0.001	1.9	0.73
Vitamin A supplementation in the last 6 months	3.3	0.03	0.2	0.93
Percentage who started breastfeeding within 1 hour of birth	20.7	<0.001	13.5	0.001
Percentage who were given colostrum	−6.1	<0.05	6.8	<0.05
Percentage who were given prelacteal feed	10.5	0.03	−17.5	<0.001
Aware about ORS preparation	−0.2	0.97	−1.3	0.69

*TT* Tetanus toxoid, *BCG* Bacille Calmette Guerin, *ORS* Oral rehydration solution.

### Health facility assessment

Contracted RHCs performed better than control RHCs in three out of five BSC domains and had a higher composite score (Table 5). Contracted RHCs had higher scores of patient satisfaction measured in terms of patient inclination to deliver at a health facility and satisfaction with the services provided. They also had better staff satisfaction levels regarding satisfaction with facility work environment and supervisory visits. Contracted RHCs also scored higher in terms of functionality as compared to control RHCs, having supplies and equipment in accordance with MNCH program guidelines for RHC, presence of waste disposal mechanism, and availability of Health Management Information System records. Contracted RHCs did not fare better than control RHCs with respect to staff capacity judged in terms of training and knowledge scores. Similarly, there was also no overall difference across contracted and control RHCs in the technical process of service provision. More specifically, antenatal assessment and communication to mothers on danger signs and newborn care were comparably similar. However, a difference in some benchmarks related to higher levels of folate prescription and carrying out of ANC-related diagnostics appeared between contracted and control sites in Chitral.

**Table 5:** **Quality of care by contracted and control rural health centres on balanced scorecard domains**

**Parameter**		**Contracted**	**Control**
	Domain A: Patient satisfaction index	(n = 20)	(n = 40)
1	Patient’s inclination for facility-based delivery	80%	43%
2	Overall patient satisfaction	100%	87%
	Total score	90%	65%
	Domain B: Staff satisfaction index	(n = 12)	(n = 24)
3	Staff satisfaction with supervisory visits	100%	53%
4	Staff satisfaction with facility work environment	92%	71%
	Total score	96%	62%
	Domain C: Staff capacity index	(n = 12)	(n = 24)
5	Staff receiving training in maternal health	91%	87%
6	Staff receiving training in newborn health	73%	47%
7	Staff knowledge score	28%	22%
	Total score	64%	52%
	Domain D: Service provision index	(n = 12)	(n = 24)
8	Communication to mothers about newborn danger signs	18%	15%
9	Communication to mothers about antenatal and post-partum danger signs	30%	26%
10	Communication to mothers about appropriate breastfeeding	50%	20%
		(n = 20)	(n = 40)
11	Women prescribed folate	70%	47%
12	Women advised about tetanus toxoid vaccine, ultrasound, and lab tests	77%	69%
13	Women provided appropriate antenatal physical assessment	20%	18%
	Total score	44%	33%
	Domain E: Health facility functionality index	(n = 2)	(n = 4)
14	Appropriate staffing	55%	58%
15	Available drugs	95%	77%
16	Available supplies and equipment	74%	60%
17	Availability of services (lab services and BEmONC signal functions)	70%	36%
18	Availability of any waste disposal mechanism	100%	50%
19	Available Health Management Information System records	80%	67%
20	Availability of any service delivery guidelines	100%	50%
	Total score	82%	57%
	Overall score	80%	60%

Key for grading system: <50%, Poor performance; 50–70%, Good performance; >70%, Excellent performance.

*BEmONC* Basic Emergency Obstetric and Neonatal Care.

### Household OOP expenditure

Clients in contracted catchments spent significantly higher amounts on transportation than those in control sites (Table 6), presumably due to the remoteness of these areas. There was also significantly higher expenditure on diagnostics, but no difference in terms of medicine and consultation. Expenditure on services, after adjusting for transport expense, was significantly lower for normal delivery and newborn illness in contracted catchments. However, expense was significantly higher on ANC visit and marginally higher for complicated delivery while there was no significant difference in caesarean delivery (Chitral only) and PNC expense in contracted as compared to control catchments.

**Table 6:** **Household mean out-of-pocket expenditure** ^**a**^ **(in PKR) by catchment population**

**Services**	**Contracted** ***(n = 350)***	**Control** ***(n = 654)***	***P*** **value**
	**Mean (SD)**	**Mean (SD)**	
Expenditure by different items			
Consultation	1428 (1310)	1427 (1110)	0.99
Medicine	912 (486)	876 (479)	0.35
Diagnostics	577 (255)	480 (258)	<0.001
Transport	2052 (1262)	1360 (1138)	<0.001
Expenditure by MNCH services (adjusted for transport expenditure)			
Antenatal care	1075 (440)	963 (443)	0.002
Normal delivery	600 (782)	1425 (1186)	<0.001
Complicated delivery	7129 (4333)	5571 (4016)	0.05
Caesarean delivery	27212 (9400)	22272 (10892)	0.18
Postnatal care	697 (414)	703 (399)	0.89
Newborn illness	746 (421)	903 (522)	0.02

^a^ Out-of-pocket expenditure data is trimmed up to 10% from both tails.

*PKR* Pakistani rupees, *MNCH* Maternal, newborn, and child health.

### Community preference for providers and barriers to utilization

FGDs held with mothers and their spouses elicited that clients residing closer to contracted RHCs preferred to seek care for childbirth and ANC from the contracted RHC, while a Dai (traditional birth attendant) was the preferred provider for childbirth amongst clients residing in the periphery of contracted RHCs and in catchment areas of control RHCs. There was common preference not to seek PNC in both contracted and control areas unless in the event of emergency and clients instead preferred to consult the Dai or LHW if available. For pregnancy complications and newborn illness, village-based care was commonly preferred to treat minor problems and only in case of serious complications respondents expressed their preference to visit RHCs or private health facilities but not government managed centres.

Clients were also probed on underlying barriers for non-usage of RHCs. Supply side concerns were more commonly reported for control RHCs and included unavailability of basic MNCH services, scarcity of medicines, inadequate attention received from staff and long waiting times. A mother from catchment of control RHC reported,

“*No facilities are available over here, neither medicines are available nor doctor pays attention. We returned back* [from RHC] *after long waiting because nobody paid attention.*” (FGD #13, P7).

For clients from contracted sites, the most formidable barrier was high OOP expenditure on transport due to remote distances. Physical access was a cross-cutting concern in both catchments but more widely prioritized by those living in catchment of contracted facilities with complaints of scarce transportation, poor road conditions, and long distances to RHCs. Respondents also cited user charges for diagnostics and registration fee for delivery at contracted facilities, which went against community perceptions of free services at government facilities. A father from catchment of contracted RHC stated,

“*In RHC Shagram, they charge 175 rupees for one X-ray. The registration fee in government hospital is 5 rupees whereas in RHC Shagram it is 75 rupees. They charge high there so we prefer Dai* [traditional birth attendant]*.*” (FGD #32, P2)*.*

## Discussion

The present study found that utilization of contracted out RHCs was significantly higher for MNCH services and the effect was seen across a range of services including ANC (3+ visits), delivery, PNC, and newborn illness. Prior contracting studies from Bangladesh, Cambodia, Bolivia, Guatemala, and India have reported only a positive increase in ANC visits, but widely variable performance for facility-based births [21-26]. The higher utilization of routine MNCH services seen in our study in the absence of performance-based incentives may be attributed to the specific focus of contracting out on MNCH services, whereas the contractual emphasis in other country contexts examined has usually been on general primary healthcare. However, there was no significant difference in population-based coverage of MNCH services by skilled provider between contracted and non-contracted catchment areas. This indicates that, while contracting out increases efficiency of the government facility through higher utilization, by itself contracting is insufficient to result in higher MNCH service coverage in contracted catchments as compared to non-contracted areas. This calls for supportive measures to accompany contracting, which are discussed below.

### Who benefits from increased access?

Equitable utilization remains under-examined. Few studies have looked into utilization of health services by disadvantaged groups, but these are not specific to MNCH services. These studies showed no difference in terms of the poor and the less poor in the use of contracted facilities in Bangladesh and Cambodia [21,26], while closer residential proximity led to better use in Guatemala [24]. Our study looked into equity aspects of utilization for a range of MNCH services and found RHC use for ANC in contracted sites to be regressively distributed towards more educated mothers and those in the higher income groups. Facility-based births, while not showing a regressive pattern, also did not show a progressive pattern indicating need for targeted outreach measures to reach the more disadvantaged.

Our study on barriers to utilization provides insight into where supportive measures may be placed. Supply side issues at contracted facilities were not a concern despite being the foremost issue at control facilities. Instead, transports costs, difficulties in physical access, and NGO-instituted user charges for registration and diagnostics remained residual barriers at contracted sites. Uneven demand for services was another factor blunting usage with little demand seen for PNC, newborn check-up, and facility-based delivery as opposed to high demand for first antenatal visit, emergency obstetric care, and newborn illness. Our study therefore indicates the need for both demand-inducing measures as well as safety nets for transport expenditure to accompany contracting. User fees applied by contracted NGOs at government facilities also need to be carefully considered as they may go against the popular notions of free services at government facilities.

### Does contracting reduce OOP expenditure?

The previous study from Cambodia reports on patient expenditure and is suggestive of reduction in contracted areas [26]. We found that increased facility utilization in contracted sites was accompanied by higher patient expense on transportation, which can be formidable in remote rural locations and requires safeguarding measures. There was also higher diagnostics-related expenditure in contracted sites as a result of budget insufficiency for the range of diagnostics agreed upon in the contract and calls for careful stipulation in contractual budget so as to prevent shifting of added expense to patients.

### Do community-based promotive practices improve with contracting?

This study also importantly highlighted that contracting out of facilities only translates into better community-based preventive care practices when control over outreach services is also provided. The contracted site lacking a control of outreach services actually had worse indicators than control sites, indicating that bifurcation of facility and outreach responsibilities may in fact have a negative effect. While little is published about preventive care impacts of contracting, two studies from Cambodia and Guatemala show a similar decrease in immunization in contracted sites [24-26]. Even with outreach control, there remained areas for further improvements, as certain services, such as tetanus toxoid immunization and knowledge of oral rehydration solution preparation, did not show any difference and may benefit from inclusion of targeted result-based payments in contracts.

### Do contracted RHCs have better quality of care?

The contracted facilities had generally better quality of care but not in all aspects. The health facility assessment shows better functionality of contracted facilities, and higher staff and patient satisfaction, but there is little edge of contracted RHCs in terms of technical process of care and staff capacities. Detailed assessments of quality of contracted primary healthcare services from Afghanistan show high scores in several but not all aspects [27]. Other studies provide evidence on improved iron supplementation [24], female client satisfaction [28], and staff presence and functionality [8] with contracting, but there is considerable variability and an absence of standardised improvements across contracting interventions. Quality of care remains a complex issue influenced by contractual incentives, control, and accountability mechanisms [29,30].

### Strengths and weaknesses

There were certain methodological limitations. Only six facilities were assessed, and although powered to detect utilization differences, our results can be interpreted as a case study that adds insight to contracting literature. The context used here is of remote rural settings and results cannot be generalizable to all settings. For quality assessment we were unable to report the technical process of care for deliveries at control sites as there were only infrequent cases. Finally, due to the absence of baseline data, we cannot establish an increase, but only a comparison of better utilization and quality of care in the contracted over control facilities.

The most visible strength of this study was that it moved beyond an assessment of facility outputs as usually performed in other studies [3], and undertook a comprehensive assessment inclusive of population-based utilization, preventive care coverage in the community, quality of care, expenditure, and client-perceived barriers. Contracted and control catchment areas were comparable in terms of education, culture, and parity with contracted areas being, if anything, more remote and having a poorer population, hence could not have contributed to the positive results. Moreover, this study undertook an in-depth assessment of a range of MNCH services, as opposed to previous studies that have either not had a focus on MNCH or reported only on ANC and delivery.

## Conclusion

Our case study from rural remote Pakistan indicates that contracting out MNCH services at government facilities to NGOs can result in significantly higher facility utilization across a range of MNCH services, better quality of certain services, and can also improve promotive care practices in the community when administrative control over outreach services is also provided as part of the contract. However, contracting in the context of rural remote settings does not result in a beneficial impact on all fronts. Higher facility utilization is confined to literate or better socioeconomic groups. Increased utilization also comes with higher client OOP expenditure on transport and diagnostic fees, and fails to translate into higher population coverage of MNCH services. For contracting to meaningfully increase access in remote settings, accompanying measures are required aimed at behavioural change measures for demand enhancement at village level, transportation access such as through incentivised transport networks or use of transport vouchers, and targeted measures for the more disadvantaged clientele.

## Abbreviations

ANC: Antenatal care
BEmONC: Basic Emergency Obstetric and Neonatal Care
BHUs: Basic Health Units
BSC: Balanced scorecard
FGDs: Focus group discussions
LHWs: Lady Health Workers
MNCH: Maternal, newborn, and child health
NGOs: Non-governmental organizations
OOP: Out-of-pocket
PNC: Postnatal care
RHCs: Rural Health Centres
